# A Case of Heterotopic Pregnancy Resulting in a Live Birth Through Laparoscopic Surgery

**DOI:** 10.7759/cureus.93544

**Published:** 2025-09-30

**Authors:** Hajime Yudate, Hiroaki Ishida, Aya Kawahara, Akiko Takashima

**Affiliations:** 1 Obstetrics and Gynecology, Toho University Medical Center Sakura, Chiba, JPN

**Keywords:** assisted reproductive technology, general anesthesia during pregnancy, heterotopic pregnancy, infertility treatment, laparoscope

## Abstract

Heterotopic pregnancy (HP) is a condition in which intrauterine and ectopic pregnancies occur simultaneously and is extremely rare. However, its incidence has markedly increased with the use of assisted reproductive technology (ART). HP can be life-threatening due to intraperitoneal bleeding; therefore, accurate diagnosis is crucial. The coexistence of intrauterine pregnancy complicates the detection of ectopic pregnancy, making HP one of the most challenging diagnoses in modern gynecological practice. Here, we describe a case in which HP was diagnosed through laparoscopic surgery, followed by a live birth. Based on a literature review, we also discussed appropriate strategies for the diagnosis and management of HP, as well as the importance of providing adequate preoperative information.

The patient was a 34-year-old, G1P0, with no significant medical history. After five cycles of timed therapy without conception, hysterosalpingography revealed severe stenosis of both fallopian tubes, and bilateral salpingoscopic salpingoplasty was performed. Oral clomiphene citrate was initiated, timing guidance was provided on day 16 after the last menstrual period, and pregnancy was established. At seven weeks and one day of pregnancy, a hematoma was detected in the left adnexal region, and she was referred to our hospital. Intrauterine pregnancy with ovarian bleeding from a corpus luteum cyst or heterotopic pregnancy (HP) was suspected. The patient and her husband initially preferred observation; however, by the third day of hospitalization, the hematoma had not reduced, and they requested surgery. For diagnostic and therapeutic purposes, laparoscopic surgery under general anesthesia was performed at seven weeks and four days of pregnancy. A diagnosis of left tubal pregnancy was made, and a left salpingectomy was performed. The pregnancy progressed smoothly, and spontaneous cephalic vaginal delivery occurred at 38 weeks and six days of gestation. The baby weighed 3,200 g at birth.

HP is extremely rare; however, its incidence has increased with the widespread use of infertility treatments. Delayed diagnosis can be life-threatening for both the mother and the intrauterine fetus; therefore, even after intrauterine pregnancy is confirmed, evaluation for HP using transvaginal ultrasound is essential. Careful management is required to preserve an intrauterine pregnancy while treating ectopic pregnancy. Patients and their families must also be provided with accurate information when laparoscopic surgery is performed under general anesthesia.

## Introduction

Heterotopic pregnancy (HP) is a condition in which intrauterine and ectopic pregnancies occur simultaneously and is estimated to occur in approximately one in 30,000 spontaneous pregnancies [1]. However, its incidence has markedly increased with the use of assisted reproductive technology (ART), ranging from 1:100 to 1:500 [2]. The risk factors for HP are very similar to those for ectopic pregnancy, including smoking, sexually transmitted diseases (especially chlamydia), tubal surgery, abdominal surgery, endometriosis, and fertility treatments [3]. HP can be life-threatening due to intraperitoneal bleeding [4]; therefore, accurate diagnosis is crucial. The coexistence of intrauterine pregnancy complicates the detection of ectopic pregnancy [4], making HP one of the most challenging diagnoses in modern gynecological practice. Additionally, there is a risk of fetal loss following surgery for ectopic pregnancy in patients with HP [5]. In recent years, however, with the growing number of HP cases associated with ART, diagnosis and treatment using laparoscopic surgery have become feasible, and increasing reports have documented successful live births [6].

Here, we describe a case in which HP was diagnosed through laparoscopic surgery, followed by a live birth. Based on a literature review, we also discussed appropriate strategies for the diagnosis and management of HP, as well as the importance of providing adequate preoperative information. In addition, in order to avoid overlooking HP, we conducted a literature review to determine what kind of management should be carried out and created a flowchart for diagnosing and treating HP.

## Case presentation

**Figure 1:**
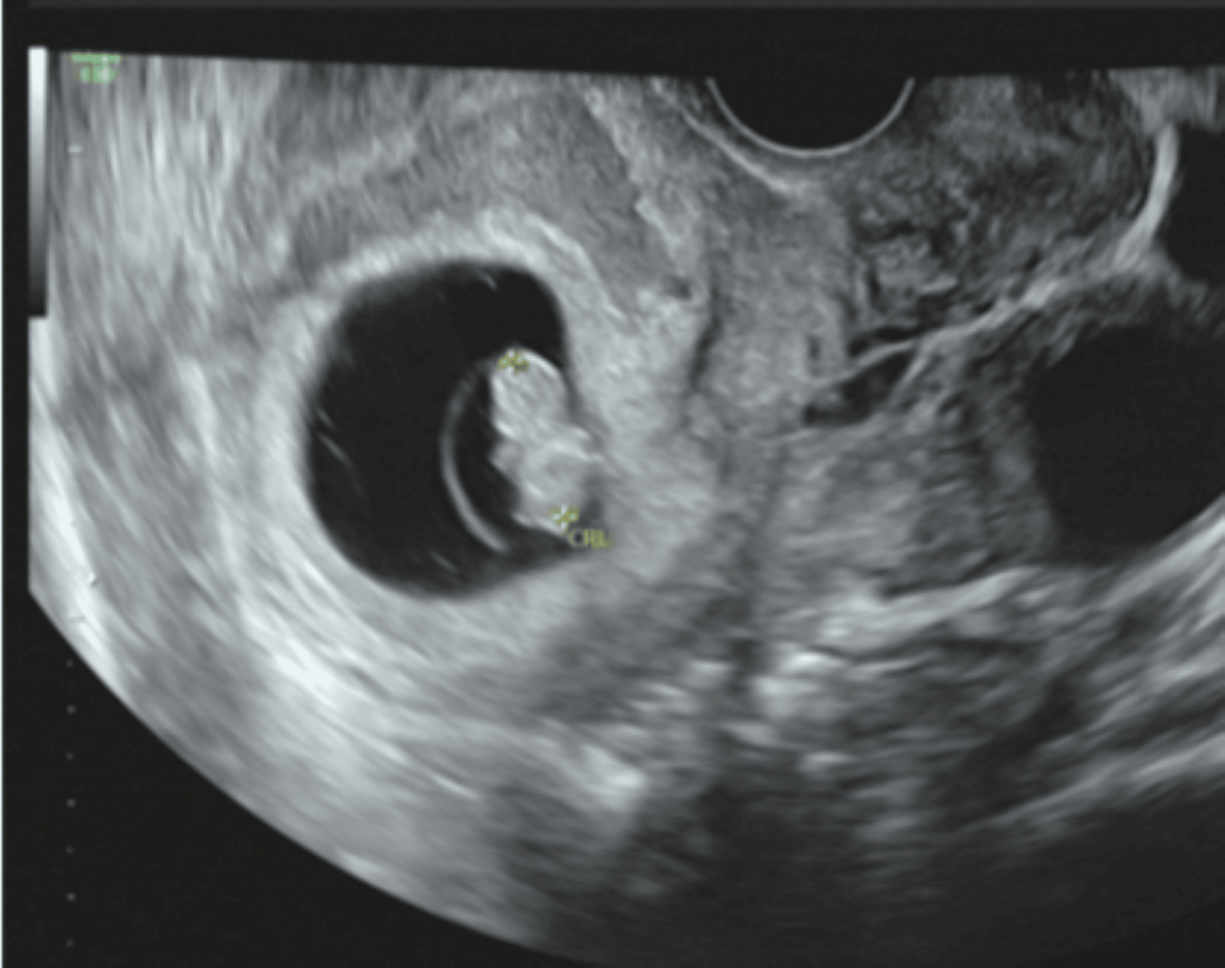
Transvaginal ultrasound. A fetus in the uterus.

**Figure 2:**
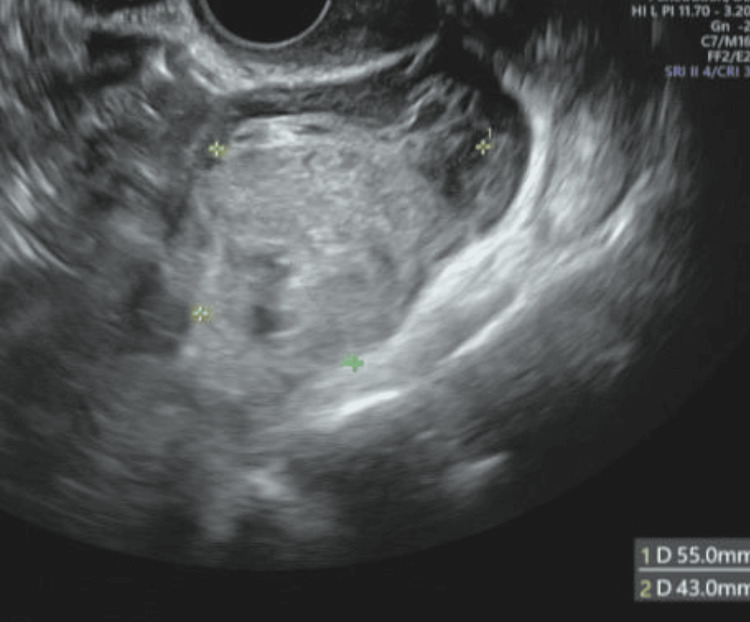
Transvaginal ultrasound. A hematoma measuring 55 mm x 43 mm around the left adnexa.

**Figure 3:**
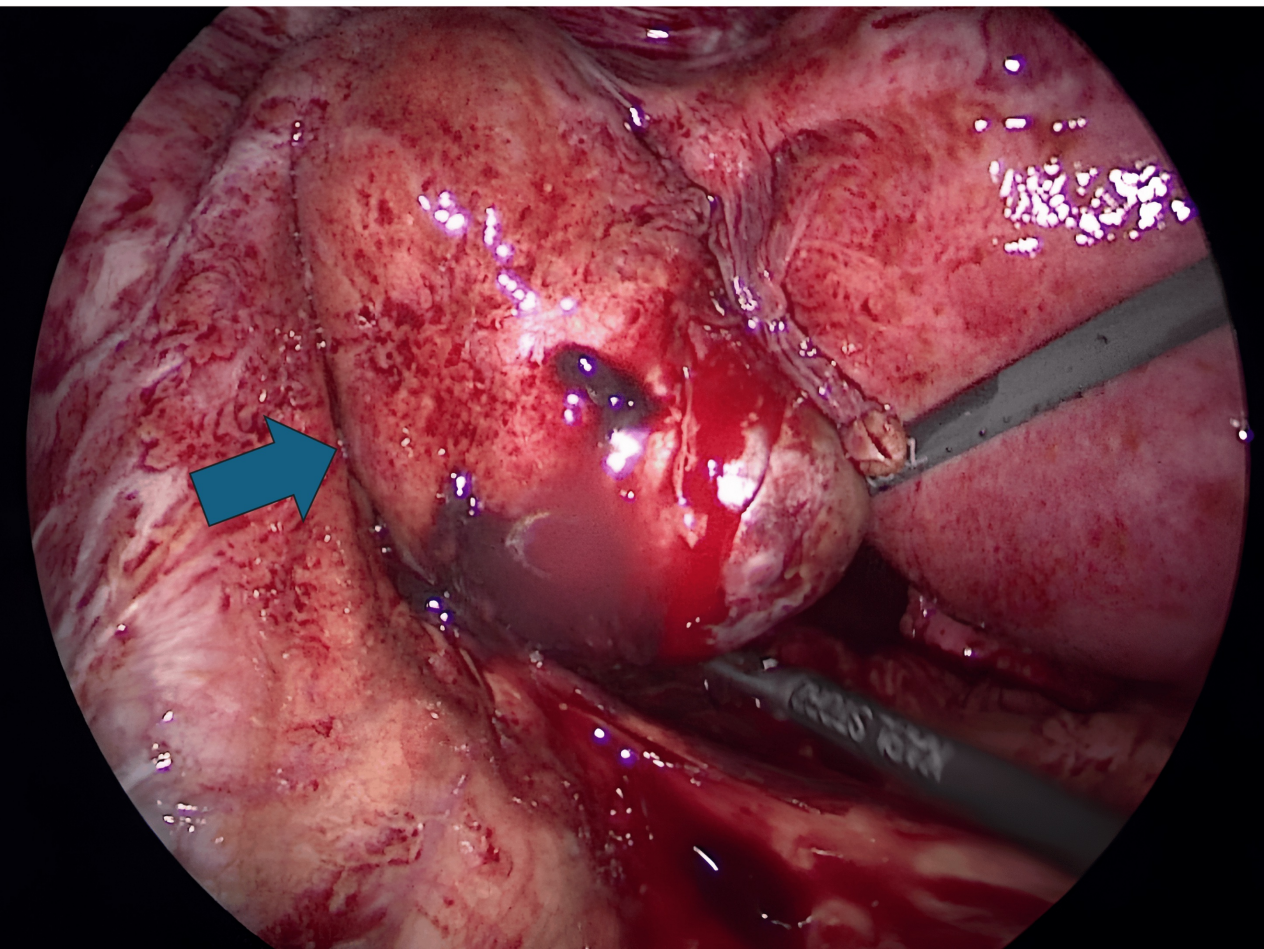
Laparoscopic finding. Swelling of the left ampulla of the fallopian tube (arrow).

The patient was a 34-year-old, G1P0, with no significant medical history. She initially visited Hospital A seeking fertility treatment. After five cycles of timed therapy without conception, she consulted Hospital B. Hysterosalpingography revealed severe stenosis of both fallopian tubes, and bilateral salpingoscopic salpingoplastia was performed. Oral clomiphene citrate was initiated, and at the time of examination, one follicle larger than 20 mm was identified in each ovary. The patient was counseled regarding the risk of multiple births, timing guidance was provided on day 16 after the last menstrual period, and pregnancy was established. At five weeks and one day of pregnancy, calculated from the last menstrual period, an intrauterine gestational sac was observed. At seven weeks and one day of pregnancy, a hematoma was detected in the left adnexal region, and she was referred from Hospital B to our hospital. At her first visit, her blood pressure was 120/60 mmHg, and her pulse was 70 beats/min, both within normal ranges, and she reported no abnormal vaginal bleeding or lower abdominal pain. Serial transvaginal ultrasound examinations revealed a fetal FHB (+) CRL of 11 mm, findings consistent with the intrauterine gestational age (Figure 1), as well as a hematoma measuring 55 × 43 mm around the left adnexa that could not be clearly identified (Figure 2). Intrauterine pregnancy with ovarian bleeding from a corpus luteum cyst or heterotopic pregnancy (HP) was suspected. We explained four points to the patient and her husband and obtained informed consent:(1) in HP, intraperitoneal bleeding may occur, which could endanger the lives of both the mother and intrauterine fetus; (2) intrauterine pregnancy may miscarry after surgery, although there is no evidence that surgery increases this risk; (3) there is no evidence that general anesthesia adversely affects intrauterine fetal growth and development; (4) laparoscopic surgery may reveal a normal intrauterine pregnancy with ovarian bleeding. Since there were no typical symptoms of ectopic pregnancy, such as abdominal pain or abnormal vaginal bleeding, the patient and her husband initially preferred observation; however, by the third day of hospitalization, the hematoma had not reduced, and they requested surgery. For diagnostic and therapeutic purposes, laparoscopic surgery under general anesthesia was performed at seven weeks and four days of pregnancy. The surgical field was secured using pneumoperitoneum with carbon dioxide, and the pelvis was raised to 10°. Because a viable intrauterine pregnancy was confirmed, a uterine manipulator was not used, and forceps manipulation was performed carefully. Laparoscopy revealed swelling of the left ampulla of the fallopian tube (Figure 3). A diagnosis of left tubal pregnancy was made, and a left salpingectomy was performed using the LigaSure™ (Medtronic, Minneapolis) Blunt Tip vessel-sealing device (Medtronic, Minneapolis). The surgery lasted 1 hour and 8 minutes, the anesthesia time was 1 hour and 49 minutes, and the blood loss was 5 g. The medications administered during surgery included remifentanil hydrochloride as an analgesic, propofol as a sedative, vecuronium bromide as a muscle relaxant, sugammadex sodium as a muscle relaxant reversal agent, and cefazolin sodium as an antibiotic. Histopathological examination of the left fallopian tube revealed chorionic tissue, confirming a definitive diagnosis of HP. The postoperative course was uneventful. Transvaginal ultrasound confirmed the continuation of intrauterine pregnancy, and the patient was discharged at eight weeks and one day of pregnancy. The pregnancy progressed smoothly, and spontaneous cephalic vaginal delivery occurred at 38 weeks and six days of gestation. The baby weighed 3,200 g at birth and had Apgar scores of 8 at 1 minute and 10 at 5 minutes. One year after delivery, there were no abnormalities in the mother and no abnormalities in the baby's growth or development.

## Discussion

HP is a condition in which an ectopic pregnancy, usually tubal, occurs simultaneously with an intrauterine pregnancy. HP is extremely rare during natural conception; however, its incidence increases with infertility treatments, particularly in vitro fertilization and embryo transfer. Risk factors include infertility treatments (especially fertility drugs and IVF), tubal factor infertility (blockage or adhesions), predisposition to multiple pregnancies (multiple ovulations induced by fertility drugs), and a history of ectopic pregnancy [3,5,7]. In the present case, the patient had a history of tubal obstruction and poor tubal patency, and ovulation-inducing drugs were thought to have caused the release of multiple eggs.

Among HP, ectopic pregnancies are most commonly tubal; however, ovarian and cervical pregnancies have also been reported. Laparoscopic surgery is often performed for intraperitoneal pregnancies, such as tubal and ovarian pregnancies, while uterine evacuation using a hysteroscope is performed for cervical pregnancies [5]. Reports indicate that even after laparoscopic or hysteroscopic surgery, intrauterine pregnancy can continue and may even reach full term [3,8].

Clinical symptoms often include lower abdominal pain and genital bleeding. However, because these symptoms can also occur frequently during normal pregnancy, it is difficult to use these symptoms as clues for early diagnosis of HP. HP is asymptomatic in 24% of cases [9,10], and this case was also asymptomatic. Abdominal pain and hypovolemic shock due to intraperitoneal bleeding are typical symptoms [9,10], and it is a potentially dangerous disease for both the mother and the fetus. Therefore, HP should always be kept in mind, even if an intrauterine pregnancy has been confirmed. A retrospective study reported that the probability of HP diagnosis during the first ultrasound examination was 64.4% [11]. Corpus luteum cysts and ovarian bleeding are considered differential diagnoses that can complicate early pregnancy diagnosis. Recent studies have shown that MRI is effective in diagnosing ectopic pregnancy [12] and is particularly useful when ultrasound findings are inconclusive. In this case, laparoscopy was performed first for diagnostic purposes; therefore, MRI was not conducted.

Conservative treatment of HP may involve monitoring the condition and waiting for hematoma shrinkage; however, in most cases, surgery is required to remove the ectopic pregnancy. Many reports describe laparoscopic surgery as the treatment of choice, but prospective studies on its safety during pregnancy are limited. Nevertheless, the 2017 American Society for Endoscopic Surgery guidelines stated that laparoscopic surgery can be performed safely during pregnancy [13]. The American College of Obstetricians and Gynecologists has also stated that there is no evidence that anesthetics affect fetal brain development and that pregnant women should not be denied necessary surgery [14]. When HP is properly diagnosed and treated, approximately 70-80% of intrauterine pregnancies survive, and postnatal outcomes are comparable to those of normal pregnancies [5]. Considering the risks of delayed diagnosis and the severity of HP due to ectopic rupture, diagnostic laparoscopic surgery is considered an effective treatment option.

**Figure 4:**
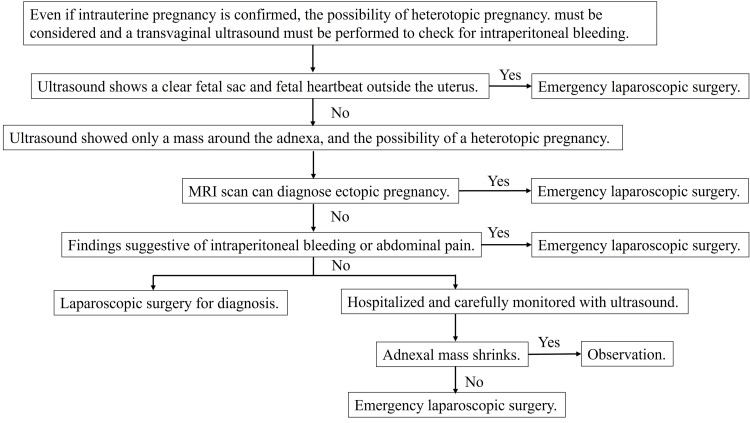
Flowchart for diagnosing heterotopic pregnancy. The image is created by the author. Ref no: [11-13]

Based on the literature review, we developed a flowchart for diagnosing HP (Figure 4) [11-13]. In this case, there was no lower abdominal pain, and ultrasonography did not reveal intraperitoneal bleeding. MRI was not performed, but as part of hospital management, the left adnexal mass was monitored regularly and showed no tendency to shrink; therefore, laparoscopic surgery was performed on the third day after admission. Table 1 summarizes the information required for obtaining informed consent from patients when diagnosing and treating HP [5,12-14]. Providing patients with appropriate diagnostic processes and evidence-based information is important for the diagnosis and management of HP.

**Table 1:** Informed consent for surgery of heterotopic pregnancy [5,12-14].

Informed consent that should be provided when performing laparoscopic surgery in suspected heterotopic pregnancy.
Heterotopic pregnancy is an acute circulatory failure caused by intraperitoneal bleeding, which can be critical for both the mother and the fetus in the uterus. Therefore, it is important to perform surgery as soon as possible to diagnose and treat the condition.
Common surgical complications (bleeding, infection, damage to nearby organs, possibility of transitioning to laparotomy).
Possibility of diagnosing ovarian bleeding rather than ectopic pregnancy.
There is no evidence that anesthetics or analgesics used during pregnancy affect fetal brain development.
Surgery should not be postponed due to potential effects on the fetus and should be performed at the appropriate time.
There is a certain probability of abortion.
There is no evidence that surgery increases the risk of abortion.

## Conclusions

HP is extremely rare; however, its incidence has increased with the widespread use of infertility treatments. Delayed diagnosis can be life-threatening for both the mother and the intrauterine fetus; therefore, even after intrauterine pregnancy is confirmed, evaluation for HP using transvaginal ultrasound is essential. Careful management is required to preserve an intrauterine pregnancy while treating ectopic pregnancy. In cases like this one, where there are no typical symptoms such as abdominal pain or abnormal bleeding, strict observation under hospitalization and the timing of laparoscopic surgery must be carefully considered. Patients and their families must also be provided with accurate information when laparoscopic surgery is performed under general anesthesia.
